# Case report: Pembrolizumab plus Axitinib related hypothyroid myopathy in two kidney cancer patients

**DOI:** 10.3389/fonc.2022.1048526

**Published:** 2022-12-02

**Authors:** Andrea Boutros, Lara Vera, Federico Gatto, Giuseppe Fornarini, Elisa Zanardi

**Affiliations:** ^1^ UO Oncologia Medica 2, IRCCS Ospedale Policlinico San Martino, Genoa, Italy; ^2^ Department of Internal Medicine and Medical Specialities, School of Medicine, University of Genoa, Genoa, Italy; ^3^ Endocrinology Unit, Department of Internal Medicine, IRCCS Policlinico San Martino, Genova, Italy; ^4^ UO Oncologia Medica 1, IRCCS Policlinico San Martino, Genova, Italy; ^5^ Department of Medical Oncology, UO Clinica di Oncologia Medica, IRCCS Ospedale Policlinico San Martino, Genoa, Italy

**Keywords:** pembrolizumab, axitinib, kidney cancer, hypothyroidism, myopathy, hypothyroid myopathy, immunotherapy, immune-relate adverse event

## Abstract

The first-line therapy in advanced kidney cancer has changed in recent years due to the introduction of combinations of tyrosine kinase inhibitors (TKIs) of vascular endothelial growth factor receptors (VEGFR) and immune checkpoint inhibitors (ICIs). Although immune-related adverse events are well-known, in the case of combination treatments, the determination of which drug is related to an adverse event may be challenging. We reported two cases of patients who developed muscle enzyme elevation in association with hypothyroidism during therapy with pembrolizumab plus axitinib for metastatic kidney cancer. The myopathy rapidly resolved after hormone replacement therapy with levothyroxine. Hypothyroid myopathy is a scarcely known and underreported adverse event. This adverse event may be relevant in the differential diagnosis with immune-related myositis, which has an autoimmune pathogenesis and a potentially fatal course.

## Background

In recent years, the therapeutic landscape for advanced, untreated kidney cancer has expanded. In particular, different combinations of immune-checkpoint inhibitors (ICIs), such as anti-programmed cell death 1 or programmed cell death ligand 1 (anti-PD-1/PD-L1) antibodies, have been studied in combination with tyrosine-kinase inhibitors (TKIs) of vascular endothelial growth factor receptors (VEGFRs), with significant benefits in terms of both progression-free survival (PFS) and overall survival (OS) (1–4). The efficacy of these combinations led to the US Food and Drug Administration (FDA) approval and widespread use of avelumab plus axitinib, pembrolizumab plus axitinib, pembrolizumab plus Lenvatinib, and nivolumab plus cabozantinib.

Although the possible adverse events, even rare ones, of both drug classes are now well-known, with the spread of these combinations, it may be challenging to recognise whether a particular adverse event is related to one or the other drug class or to both. Moreover, adverse events related to these combinations are often not reported.

We present two cases of patients with metastatic kidney cancer treated with first-line pembrolizumab plus axitinib combination, who simultaneously developed asymptomatic dysthyroidism and creatine phosphokinase (CPK) elevation.

We also conducted a systematic literature search to improve our understanding of these adverse events (sometimes even with an asymptomatic course) during treatment with pembrolizumab plus axitinib.

## Case reports

### Case 1

A 52-year-old female patient with no relevant medical history, except for allergy to erythromycin and acetylsalicylic acid, was admitted to the Emergency Department in July 2021 for haematuria. The CT scan showed a 90 × 85 mm left kidney mass, bilateral lung, and abdominal lymph node lesions.

**Figure 1:**
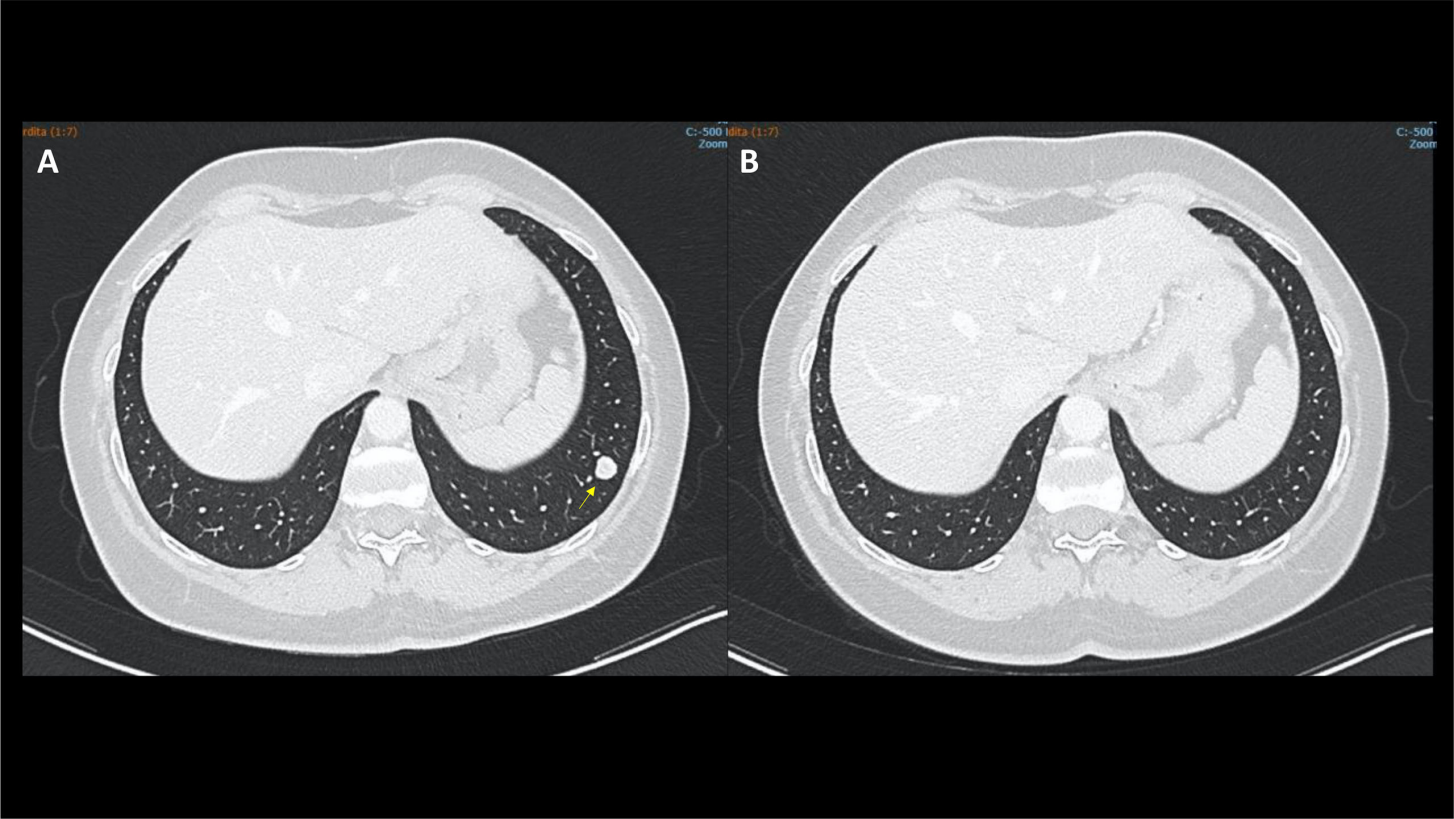
Case 1, CT scan showing a significant shrinking in the left lung lower lobe (arrows) from December 2021 **(A)** to May 2022 **(B)**.

After multidisciplinary discussion, a cytoreductive video laparoscopic left nephrectomy was performed, with histological diagnosis of grade 3 clear cell renal cell carcinoma extending to the fatty tissue of the renal sinus. Excision margins were not involved by the tumour. Due to the presence of highly suspicious pulmonary nodules (**Figure 1**), the case was discussed again after surgery with the multidisciplinary team in July 2021, with a decision of watchful waiting.

In December 2021, a new CT scan showed a dimensional increase in the left lung lower lobe (15 × 12 mm) and the appearance of a metastatic nodule in the pancreatic head (6 mm). The patient, identified as having intermediate risk according to the International mRCC Database Consortium (IMDC) criteria due to the presence of metastases at diagnosis of renal cell carcinoma (RCC), was then started on pembrolizumab 200 mg every 3 weeks plus axitinib 5 mg twice daily in January 2022. In February 2022, the patient complained of anterior neck pain and swelling. Laboratory tests showed transient hyperthyroidism (**Table 1**), which was treated with tapazole. In March 2022, there was also a grade 2 increase in transaminases, with aspartate aminotransferase (AST) level of 90 U/L and alanine aminotransferase (ALT) level of 141 U/L according to the Common Terminology Criteria for Adverse Events (CTCAE) and grade 2 increase in CPK (400 U/L). Therefore, therapy with pembrolizumab plus axitinib was withdrawn, and prednisone 10 mg daily was started until complete resolution of the symptoms and normalisation of AST, ALT, and CPK levels. Residual hypothyroidism was treated with hormone replacement therapy with levothyroxine. In April 2022, the patient was restarted on pembrolizumab 200 mg every 3 weeks. The dose of axitinib was reduced to 6 mg daily due to the occurrence of grade 2 hypertension.

**Table 1:** Thyroid hormones, transaminases, and creatine phosphokinase levels over time.

Case 1							
Date	09/02/2022	02/03/2022	17/03/2022	12/04/2022	26/05/2022	16/06/2022	27/07/2022
**AST**	32	101	90	34	33	31	28
**ALT**	54	153	141	32	38	30	24
**CPK**	117	484	705	440	315	355	269
**TSH**	0.099	106.300	150.700	129.000	48.330	41.360	18.400
**fT4**	9.50	1.81	2.86	7.28	11.36	12.81	13.31
**Case 2**							
Date	18/01/2022	10/02/2022	16/02/2022	24/02/2022	15/03/2022	05/04/2022	19/07/2022
**CPK**	85	263	467	637	192	101	125
**TSH**	34.370	205.000	196.500	194.000	141.500	49.230	35.100
**fT4**	5.12	1.54	2.48	3.69	6.97	10.90	10.73

ALT, alanine aminotransferase, normal range of 0–40 U/L; AST, aspartate aminotransferase, normal range of 0–40 U/L; CPK, creatine phosphokinase, normal range 26–170 U/L; fT4, free thyroxine, normal range of 9.30–17.00 ng/L; TSH, thyroid-stimulating hormone, normal range of 0.270–4.200 mUI/L.

At the CT reassessment in May 2022, a partial remission of the disease was observed, and the combination therapy is still ongoing.

### Case 2

A 55-year-old male patient with a history of heavy smoking (40 pack/year) and HCV-related hepatitis (eradicated in 2018) underwent a left nephrectomy in 2018 for a pT1b pN0 grade 3 clear cell renal cell carcinoma. During follow-up, in December 2021, a CT scan showed a suspicious bone remodelling area in the left clavicle and abnormal axillary and mediastinal lymph nodes (**Figure 1**). A bone biopsy reported a bone metastasis from a clear cell renal cell carcinoma.

The patient, identified as having good risk according to IMDC criteria, was then started on pembrolizumab 200 mg every 3 weeks plus axitinib 5 mg twice daily in December 2021. Treatment was stopped as precaution in February 2022 after the onset of asymptomatic hypothyroidism and CPK increase (263 U/L), as shown in **Table 1**. However, CPK continued to rise (603 U/L), in the absence of any symptom, so the patient was referred to a cardiologist who found no cardiac disorders. Autoimmunity tests showed high levels of anti-thyreoperoxidase antibodies (TPOs) (534 U/mL [normal range < 30]).

**Figure 2:**
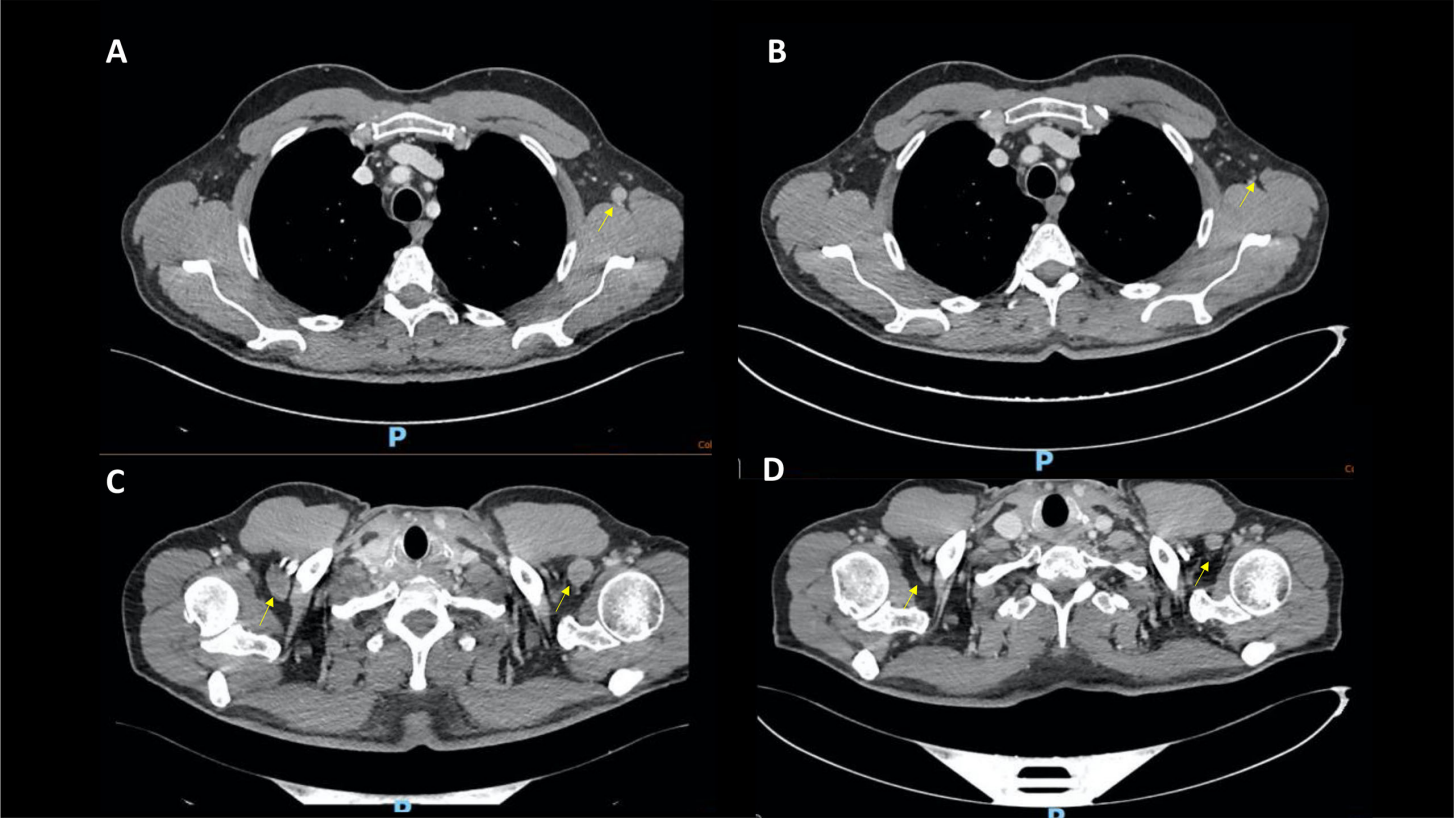
Case 2, CT scans showing significant shrinking in the axillary lymph nodes from December 2021 **(A, C)** to April 2022 **(B, D)**.

He was then started on levothyroxine and prednisone 50 mg daily, tapered until resolution of CPK elevation. The patient has then been resumed to pembrolizumab plus axitinib in March 2022. At the CT reassessment in April 2022, the left clavicle skeletal metastasis remained stable with a substantial reduction in the axillary lymph node (**Figure 2**). The patient is, therefore, continuing the current treatment.

## Discussion

The combination of ICI and TKI is being increasingly used in the first-line treatment of advanced kidney cancer (5).

We presented two cases of occurrence of thyroid dysfunction and mild muscle toxicity during therapy with pembrolizumab and axitinib, both resulting in a favourable outcome. To increase our knowledge on this adverse event, we performed a systematic literature search on 26 July 2022. We performed a computerised search through the PubMed search engine with the string (“pembrolizumab”[Title/Abstract]) AND (“axitinib”[Title/Abstract]), finding one case of myasthenia gravis, primary adrenal insufficiency, and progressive hypothyroidism, which occurred during therapy with pembrolizumab plus axitinib (6). However, both of the cases that we have presented were characterised by asymptomatic or paucisymptomatic CPK elevation (with or without elevation of transaminase levels) associated with dysthyroidism (sometimes symptomatic with signs of local inflammation).

Although cases of muscle toxicity related to pembrolizumab and other ICIs have already been reported, these are often characterised by a tumultuous, acute course, symptomatic for myalgias and muscle weakness, frequently associated with myasthenia gravis and myocarditis, and a dismal prognosis (7). On the other hand, there are few data on the significant elevation of muscle enzymes with a favourable or self-limiting course.

Both of the reported cases are representative of a clinical entity described in the literature as hypothyroid myopathy (8). The diagnostic hypothesis of hypothyroid myopathy was supported by the normalization of the CPK levels with the thyroid hormone supplementation. Hypothyroid myopathy is a lesser-known and under-reported adverse event (8), which, however, may be relevant especially during the diagnostic workup when an immune-related myositis is suspected. Expanding the literature search to include all potential cases of hypothyroid myopathy (even in patients with different tumour types, even receiving different ICIs), we found the case of a 47-year-old female patient with advanced nasopharyngeal carcinoma treated with the anti-PD-1 camrelizumab (9). The patient manifested myalgias with a marked elevation of muscle enzymes and transaminases, associated with hypothyroidism (9). All clinical muscular manifestations resolved after hormone replacement therapy with levothyroxine (9). The patient also resumed camrelizumab treatment after a short discontinuation period, with a progression-free survival of 9 months (9).

According to the American Thyroid Association guidelines, referring to the general population, an increase in serum muscle enzymes or lactate dehydrogenase (LDH) persisting for at least 2 weeks is enough to require TSH to confirm or exclude hypothyroidism (8).

The effects of hormone deficiency may affect the peripheral nervous system at any of the possible levels: nerve, neuromuscular junction, and muscle fibres (8).

The skeletal muscle expresses type 2 deiodinase, and through this enzyme, it obtains triiodothyronine (T3) from thyroxine (T4). With an adequate amount of T3, the muscle is able to perform the physiological contraction–relaxation process of the muscle fibre (8). Hypothyroid myopathy can therefore develop through several mechanisms, which are still poorly known. It can be the result of a combination of the following:

1. Alteration of glycogen metabolism and oxidative phosphorylation. T3 is associated with the upregulation of several genes involved in the citric acid cycle. Being dependent on glycolysis for energy, type 2 muscle fibres slow their contractile muscle capacity. During hypothyroidism, the reduction in glycogenolytic activity results in the accumulation of glycogen deposits. The accumulation of glycosaminoglycans and connective tissue can cause muscle hypertrophy (10). These deposits disappear once euthyroidism has been restored (11).
2. Alteration of the actin–myosin unit. T3 affects the transcription of genes involved in the expression of myosin heavy chain, which is a component of myofibrils (8).
3. A reduction in muscle carnitine levels in patients with thyroid dysfunction leading to myopathic manifestations (10).

Finally, TSH does not appear to play a role in thyroid myopathy. As also demonstrated in the reported cases, CPK levels dropped earlier than TSH levels after hormone supplementation. In fact, TSH receptors, except for the extrinsic muscles of the eye, were not expressed in skeletal muscles (8).

This metabolic-based aetiopathogenetic mechanism is different from the autoimmune and inflammatory mechanism underlying immune-related, and better known, myositis.

With the spread of indications for the use of ICIs, it becomes relevant to know even the rarest forms of drug-related myopathies. In fact, the clinical management of the two entities is extremely different. Inflammatory myositis requires aggressive immunosuppressive treatment, leading in most cases to the definitive discontinuation of immunotherapy. Thyroid myopathy, on the other hand, resolves with hormone replacement therapy, allowing the resumption of ICIs treatment (9).

## Conclusions

With the increasing indications of both ICIs and TKIs, both in combination and in monotherapy, awareness of even the rarest adverse events is essential. Hypothyroid myopathy is a little-known clinical entity characterised by a significant elevation of muscle enzymes secondary to hypothyroidism. Its clinical course is favourable and spontaneously responding to hormone therapy with levothyroxine.

## Data availability statement

The raw data supporting the conclusions of this article will be made available by the authors, without undue reservation.

## Ethics statement

Written informed consent was obtained from the individual(s) for the publication of any potentially identifiable images or data included in this article.

## Author contributions

AB: article writing and review of literature. EZ, LV and FG: patient management, diagnostic and therapeutic workup. EZ and GF supervised this work. All authors contributed to the article and approved the submitted version.

## Conflict of interest

The authors declare that the research was conducted in the absence of any commercial or financial relationships that could be construed as a potential conflict of interest.

## Publisher’s note

All claims expressed in this article are solely those of the authors and do not necessarily represent those of their affiliated organizations, or those of the publisher, the editors and the reviewers. Any product that may be evaluated in this article, or claim that may be made by its manufacturer, is not guaranteed or endorsed by the publisher.
